# Chromatic Confocal Displacement Sensor with Optimized Dispersion Probe and Modified Centroid Peak Extraction Algorithm

**DOI:** 10.3390/s19163592

**Published:** 2019-08-18

**Authors:** Jiao Bai, Xinghui Li, Xiaohao Wang, Qian Zhou, Kai Ni

**Affiliations:** 1 Division of Advanced Manufacturing, Graduate School at Shenzhen, Tsinghua University, Shenzhen 518055, China; 2 Institute of Materials, China Academy of Engineering Physics, Mianyang 621907, China

**Keywords:** chromatic confocal technology, dispersion probe, modified centroid peak extraction algorithm, calibration experiment, Fresnel lens

## Abstract

Chromatic confocal technology (CCT) is one of the most promising methods for the contactless and accurate measurement of structure profiles. Based on the principles of chromatic dispersion and confocal theory, a dispersion probe is proposed and optimized with several commercial and cheap refractive index lenses. The probe provides 0.3× magnification and a dispersion range of 400 μm with a commercial LED source with an effective bandwidth of ca. 450–623 nm. Since the noise fluctuation can affect the extraction stability of the focal wavelength, a modification to the centroid peak extraction algorithm is proposed in this paper, where several virtual pixels are interpolated among the real pixels of the spectrometer before thresholding. In addition, a series of experiments were carried out to test the system’s displacement measurement performance. The results clearly show that stability is improved by the modified algorithm, and the calibration repeatability is ±0.3 μm in the full measurement range with a linear stage. The standard deviation at the fixed position has an optimal value of 0.009 μm. The section profile of a Fresnel lens is measured by the CCT system to demonstrate its high feasibility and efficiency.

## 1. Introduction

Since chromatic confocal technology (CCT) was presented, it has become one of the most useful and potential methods for the displacement measuring. As noncontact CCT systems provide axial distance information using the dispersion lens, most structural dimensions can be determined by scanning the sample’s surface [1,2,3]. Compared to other conventional techniques for displacement, distance or position [4,5], CCT has unique advantages in some measuring applications. Firstly, CCT belongs to the noncontact methods without contacting the sample, avoiding harm to the soft or coated surface. Sometimes, the contact probe cannot reach the narrow corners in the coordinate measuring machine (CMM) or stylus profiler [6,7]. By contrast, the optical sensors can help to provide better solutions for these problems, such as laser triangulation, laser interferometry, grating interferometry, and CCT, to name but a few [8,9,10]. Laser triangulation is popular in many commercial applications with relatively high accuracy and ability to be integrated. It is, however, limited to the measurement of highly reflective or tilted surface, and the defocusing spot increases sharply around the focus position, which will lead to the decrease of lateral resolution. Laser interferometry possesses high performance with linear incremental measurements for a reflective surface fixed on the moving targets. However, laser interferometry is strictly vulnerable to environmental variables, such as air pressure, temperature, and installing space. Similarly, grating interferometry needs to install a scale grating on the traveling target to be measured. Obviously, these optical methods are mainly supported by the high-energy laser, which is sometimes dangerous to the operators. CCT has been attracting increasing attention in recent years due to its high suitability in the abovementioned situations with safe and long-lifetime LED (light emitting diode) light source. It utilizes both dispersion phenomenon and confocal technology to achieve focal position by analyzing the reflected light spectrum. The advantages can be concluded as follows: noncontact measuring mode, suitability for big tilt surface, small focal spot, high accuracy, and flexible integration without extra accessories. In addition, CCT avoids the axial scanning operation to carry out the measurement easily in many online applications, which is very tough for laser scanning confocal microscopy [11,12]. With these general performance and measuring capabilities, CCT is playing increasingly important roles in academia and industry [13,14,15].

Usually, a CCT system contains four parts: a LED light source, dispersion probe, fiber coupler, and spectrometer. LED is often used to provide white light to generate various axial foci using the dispersion probe. The fiber coupler is becoming an increasingly popular method to deliver LED light to the dispersion probe and receive the reflected light from the sample surface, i.e., it plays the roles of the beam splitter and the pinhole. Finally, the spectrometer is used to analyze the reflected light and determine the focal wavelength which is directly related to the distance from the probe. The benefits of the sensitive photoelectric response of the spectrometer is that the CCT measuring process has a faster response, usually within a few milliseconds, allowing efficient matching of high scanning speeds or vibration frequencies.

Among the above components, the dispersion probe plays the most important role as it modulates the inlet white light for axial dispersion, which realizes axial position coding for the focal wavelength. The common dispersion probe is a series of lenses that focus light on the sample and focus reflected light to the detection fiber port. The probe’s dispersion property will directly determine the measuring range, while the size of the focus point should be as small as possible in order to increase lateral resolution. The probe’s numerical aperture (NA) is also an essential characteristic which determines the inclination of the surface that can be measured effectively.

In previous studies, various types of optical designs were proposed to realize the unique requirements of the dispersion probe. In contrast to the traditional optical design of microscope objectives, the design objective is not to decrease but to increase the axial dispersion aberration. Minoni [16] used a single CaF_2_ lens to generate dispersive light with a 280 μm axial chromatic aberration from 500 to 900 nm. Kim [17] employed several lenses to achieve a 105 μm axial chromatic aberration, while Jiao [18] studied the performance of a CCT system with 1005 μm dispersion range using a set of the glass lens. Similarly, Molesini [19] utilized commercial microscope objectives to focus light on the surface. Shi [20] adopted two singlet lenses and a 100× microscope objective to achieve a dispersion range of only 8 μm. Besides these refractive index lenses, diffractive elements have also been introduced as the dispersion part in many CCT studies. Dobson [21] used a diffractive lens and microscope objectives to achieve 55 μm dispersion. Rayer [22] employed a hybrid aspheric diffractive lens to broaden the dispersion range twice. In comparison, ordinary refractive index lenses allow more light to pass through than the diffractive lenses, which is beneficial for the signal-to-noise ratio (SNR) of the measuring system. In addition, the optical design with ordinary refractive index lenses is, to some extent, more flexible, allowing multiple simultaneous targeting. However, it could be very expensive to bring out the dispersion probe with the customized lenses in the flexible optical design. Therefore, this paper proposes a feasible optical design with several commercial refractive index lenses optimized using the optical simulation software (ZEMAX). We described the detail optical design process and discussed the theoretical assessment elements, which has rarely been done in previous research.

Besides the optimal dispersion probe, peak extraction also plays an important role in enhancing measuring accuracy. Since CCT has been developed from laser scanning confocal microscopes (LSCMs) with confocal signals, they have a similar method of peak extraction. Theoretically, when an LSCM scans vertically at a certain rate, the intensity of the reflected light will form a regular curve like sinc^2^. Based on this phenomenon, Tan [23] developed a sinc^2^ fitting algorithm to achieve a reliable and accurate method for height extraction in surface topography measurements. Cheng [24] proposed a parabolic fitting algorithm for peak extraction from axial response signals. However, in the CCT systems, the reflected spectrum is the main signal to be analyzed without a scanning operation. Hence, the light intensity distribution on the optical axis is no longer following the curve of sinc^2^, and the reflected spectrum is often broader and more irregular than that of LSCM. Hence, we employed the centroid algorithm to extract the focal wavelength of the irregular reflected spectrum, which is another popular strategy for peak extraction besides the abovementioned fitting algorithm [25]. In the centroid algorithm, the abscissa value of the signal is equivalent to the vector of the particle system, and the ordinate value is equivalent to the mass of the particle system. Then, the center of mass is maintained as the focal coordinate, which is usually simple and efficient without complex computation of the optimal approximation.

Thresholding is usually recommended before applying the centroid algorithm to decrease the harm of the unsatisfactory reflected spectrum, modulated and affected by the light source, the dispersion probe, and even the fiber transmittance. Meanwhile, the intensity beside the peak of the reflected spectrum is always weak and can be easily influenced by the photoelectricity noise in the spectrometer. Hence, the SNR of the reflected spectrum is always too poor to extract an accurate focal wavelength. To enhance the stability of the centroid algorithm, we proposed a strategy whereby several virtual pixels are interpolated among the real pixels of the spectrometer’s linear array CCD before thresholding. Here, we used the pixel serial number to represent the wavelength based on the detecting principle of the spectrometer. In this way, the pixel density is increased, lowering the weight of the valid data near the threshold in the centroid algorithm.

Finally, calibration and stability experiments were conducted to test the performance of the optical design and the amended algorithm for displacement measurement. Furthermore, a Fresnel lens is measured with the optimized CCT system. The result shows great potential for high-speed surface profiling of microstructures.

## 2. System Design

**Figure 1:**
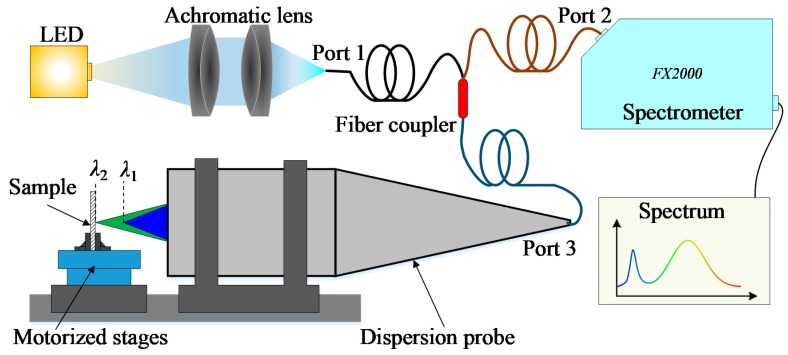
Schematic diagram of the chromatic confocal technology (CCT) system.

The schematic diagram of our CCT system is shown in Figure 1. A commercial LED light source (Cree, Inc. Durham, NC, USA) generates broad-spectrum white light at about 400–800 nm. Then, we used two achromatic lenses (Unionoptics, Inc. Wuhan, China) to collimate and focus the white light into fiber port 1, whose core diameter of 50 μm is small enough to act as the first pinhole. Passing through the fiber coupler (Thorlabs, Inc., TM50R5S1A, Newton, NJ, USA) with a splitter ratio of 50:50, most of the light is emitted from fiber port 3 with a certain numerical aperture, while some of the light will be used as the noise signal in the spectrometer (Ideaoptics, Inc., FX2000 Shanghai, China). The dispersion probe transmits white light and focuses monochromatic light at a different axial position because of the dispersion aberration of the lens. The light reflected from the sample surface returns into the optical probe and fiber couple port 3, which plays the role of the detection pinhole. As previously, the fiber coupler will split light into two parts through ports 1 and 2. The spectrometer will accept the reflected light from port 2 and analyze the spectrum. In this system, traditional pinholes are replaced by the fiber cores to meet the confocal requirement, while the dispersion probe creates a one-to-one relationship between wavelengths and distances. For example, only the focal wavelength *λ*_2_ can be reflected to port 3 and corresponds to the distance between the fixed dispersion probe and the sample surface. The sample is fixed onto motorized stages to move the orthogonal axis for experimental measurements.

**Figure 2:**
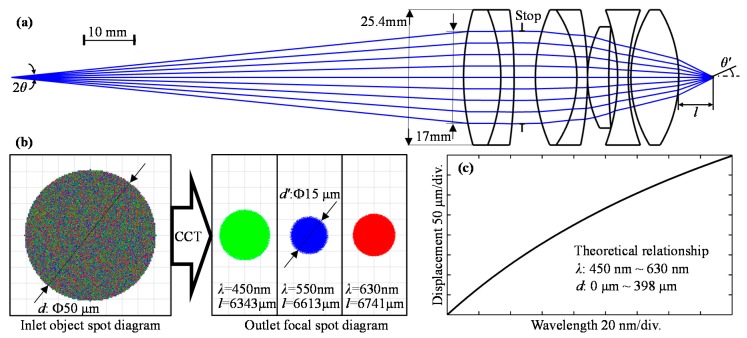
The optical design of dispersion probe: (**a**) Light path at the design wavelength 550 nm; (**b**) The spot diagram diameter change by the CCT probe; (**c**) Theoretical relationship between the wavelength and the displacement.

The dispersion probe is the key component that affects the measurement range, resolution, and accuracy. We designed the optical system of the dispersion probe in Figure 2a using several refractive index lenses.

Based on optical theory, the magnification *μ* can be expressed in the below formula with the following parameters. *NA* is the numerical aperture in the object space. *NA*’ is the numerical aperture in the image space. The inlet object spot diagram diameter is *d*, and the outlet focal spot diagram diameter is *d*’.

(1) $$\mu =\frac{NA}{NA^{\prime }}=\frac{d^{\prime }}{d}$$

To reflect as much of the focal wavelength light as possible and block off defocused light of other wavelengths, the outlet focal spot diagram diameter of the focal wavelength should be small enough and *NA’* should be as large as possible. On the other hand, an *NA’* that is too large will also increase lateral aberration and decrease the dispersion range. Here, we chose 0.3–0.4 as *NA’* in the paper. Considering the lateral resolution, we would like to make *d’* smaller than 20 μm, which is enough for most Fresnel lenses. The inlet object spot diagram diameter *d* is decided by half of the core diameter, namely, 50 μm. Thus, magnification *μ* should be smaller than 20/50 = 0.4. Hence, we find that the effective *NA* is about *μ × NA’* = 0.12–0.16, which is smaller than the fiber’s intrinsic *NA* of 0.22, resulting in losing part of the light from the entire fiber port. Several refractive index lenses were selected and simulated in ZEMAX. By optimizing the optical path with multiple targets of good imaging quality and a large dispersion range, a suitable optical design was determined and is shown in Figure 2a. The total distance from the inlet object to the focal point is about 130 mm. The inlet aperture on the first lens surface is limited to about 17 mm by the stop, and the diameter of the refractive index lenses is 25.4 mm.

For the design wavelength 550 nm, we simulated the image spots of three different wavelengths after the dispersion probe in Figure 2b. It can be concluded that the 50 μm diameter light source generates a spot with a geometrical diameter of about 15 μm, i.e., the magnification μ is about 15/50 = 0.3. The focal length *l* varies with different wavelengths. From ZEMAX, we also obtained half of the aperture angle *θ’* to be 26.94°, so *NA’* in the image space is about sin 26.94° = 0.45. According to Equation (1) above, *NA* in the object space is about 0.135, which means that part of the incident light is blocked out of the optical system. The energy loss is acceptable for the measurement because the distance is only determined by the reflected light spectrum, rather than light intensity. In addition, the LED light source can be adjusted to be brighter or darker to make the reflected light intensity suitable for detection by the spectrometer.

Finally, the theoretical relationship between the displacement and the wavelength is shown in Figure 2c as a nonlinear curve. The displacement range is 398 μm with the wavelength bandwidth ranging from 450 to 630 nm.

## 3. Modified Peak Extraction Strategy

The distance from the dispersion probe to the sample surface has a one-to-one relationship with the focal wavelength. To extract the focal wavelength, the reflected light is firstly separated by a triangular prism or a concave grating and detected on the pixels of the linear array CCD in the spectrometer. Hence, the pixel serial number will directly correspond to the wavelength. As a result, the distance also has a one-to-one relationship with the pixel serial number.

**Figure 3:**
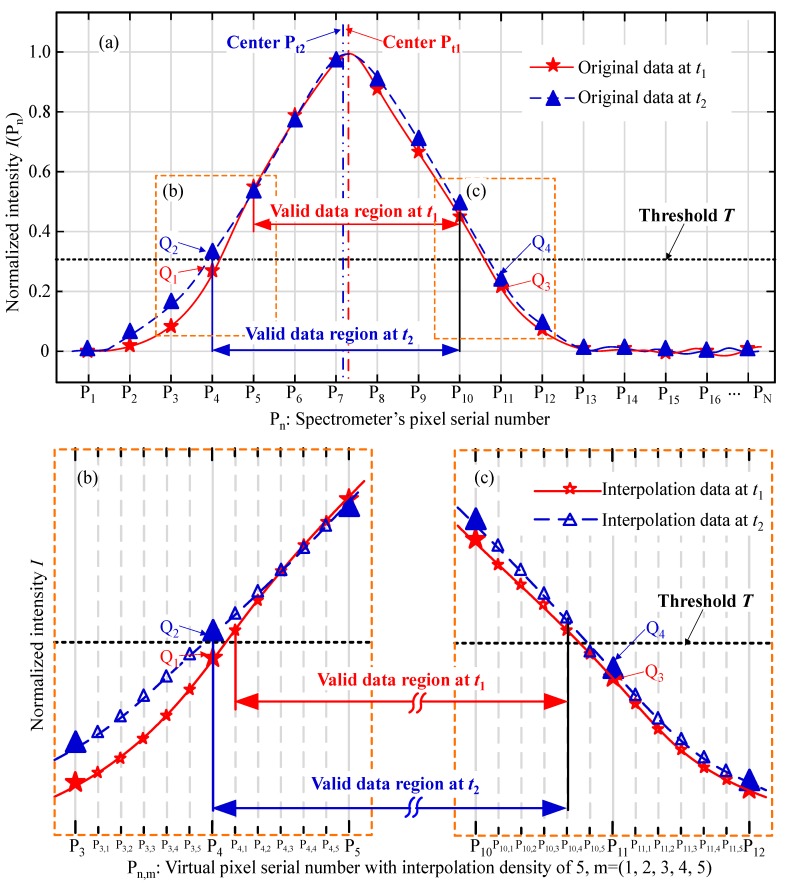
Schematic of the thresholding peak extraction algorithm.

Since the pixels are separated from each other at a certain interval, the spectrum is always discrete, with each pixel returning independent data. As we know, the intensity of the LED source fluctuates over time, and noise usually occurs during photoelectric detection, causing spectrum instability. In Figure 3, two spectra at different time *t*_1_ and *t*_2_ are shown with several original datapoints obtained at different pixels. To explain the strategy in simple terms, we suppose the total pixel number is P_N,_ and the peak mainly spreads from P_0_ to P_16_. Due to the existence of low-intensity noise, an empirical threshold *T* is usually applied before signal processing [26]. Only the data above *T* will take part in the computation of the centroid algorithm, which is used in this paper to extract the focal wavelength. Although the value of *T* cannot be exactly defined based only on the signal, we think it should be balanced in the practical processing. In the principle of the confocal technology, the ideal signal should be as narrow as possible to obtain an exact focal wavelength. That is to say, most of the defocus data is usually thought to be invalid for the peak extraction algorithm. Hence, the threshold should not be set so low as to allow more defocusing data into the calculation. On the other hand, if the threshold gets too close to the peak, there are few valid data left in the calculation, probably causing serious instability of the focal center. Thus, we think the value should be selected according to the SNR of the signal and less than half of the max intensity. In this paper, we simply define the value to be 0.3 as an example to explain the modified centroid algorithm.

As the spectrum fluctuates over time, some points may cross the threshold. Figure 3 shows an example of a common situation. Q_1_, Q_2_, Q_3_, and Q_4_ are four points near the intersection of the spectrum and the threshold. At pixel P_4_, Q_2_ is above the threshold, so it is included in the calculations, while Q_1_ is below the threshold. Meanwhile, Q_3_ and Q_4_ at pixel number P_1__1_ are both below the threshold and will not affect the centroid algorithm. This means that the fixed threshold would change the quantity of the valid data in the calculation as below.

(2) $$P_{t1}=\frac{\sum_{n=5}^{n=10}P_{n}I(P_{n})}{\sum_{n=5}^{n=10}I(P_{n})},\;P_{t2}=\frac{\sum_{n=4}^{n=10}P_{n}I(P_{n})}{\sum_{n=4}^{n=10}I(P_{n})}$$

Namely, the mass center P_t1_ can be obtained with a valid data region from P_5_ to P_10_, while P_t2_ is achieved from P_4_ to P_10_. Hence, we can conclude that a peak extraction error occurs between *t*_1_ and *t*_2_, mainly because of the addition of the datapoint Q_2_. To decrease the error’s influence, we propose a modified centroid method with several virtual pixels interpolated among the real pixels, which are shown in Figure 3b,c. Here, we take the interpolation density of 5 to explain the interpolation procedure. Between the real pixel P_n_, 5 equidistant virtual pixels are interpolated at the fitting curve with the original data. By interpolating the virtual pixels, the valid data region at *t*_1_ is changed from P_4,1_ to P_10,4_, while the valid data region at *t*_2_ is from P_4_ to P_10,4_. Thus, the difference between the valid data region at *t*_1_ and *t*_2_ is narrowed to within one-fifth of the real pixel interval, reducing the calculation weight of the fluctuating data at P_4_.

**Figure 4:**
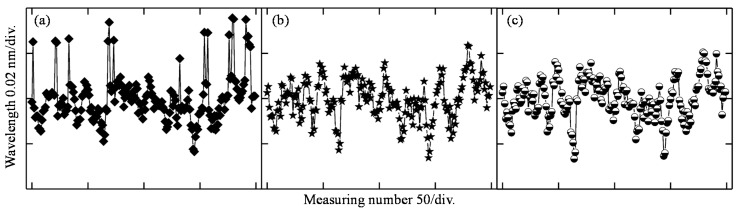
Wavelength fluctuation (**a**) with the centroid method, (**b**) with the modified centroid method by the interpolation density of 5, and (**c**) with the modified centroid method by the interpolation density of 9.

In order to evaluate the performance of the modified centroid method, we conducted a series of experiments. The mirror surface was fixed in the middle of the measuring range, and then the reflected light spectrum was obtained for multiple times with a time interval of one second. The total measuring number is 200×. After subtracting the noise signal and cutoff by a threshold *T* (0.3), the signals were processed by the centroid method and the modified centroid method with interpolation densities of 5 and 9. The results of the extracted focal wavelengths are shown in Figure 4, which shows that the range of the fluctuation with the centroid method is about 0.059 nm and the standard deviation is 0.011 nm. The corresponding values in Figure 4b,c show smaller fluctuations, with ranges of 0.050 and 0.047 nm, and standard derivations of 0.0091 and 0.0089 nm, respectively. That is to say, the modified centroid method will suppress fluctuation, improving measurement resolution and stability.

**Figure 5:**
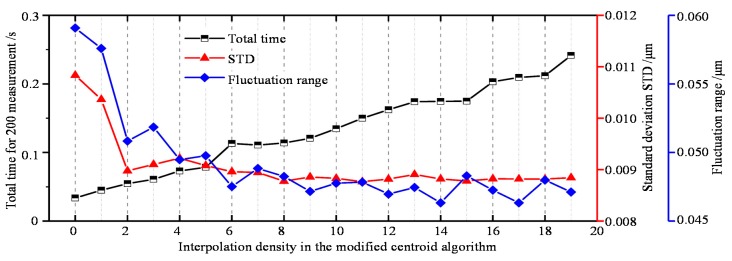
General performance of the centroid algorithm with different interpolation densities.

Furthermore, we studied the general performance of the modified centroid algorithm with different interpolation densities in Figure 5. Particularly, the total time, STD, and fluctuation range are chosen as the test metrics. This shows that higher interpolation density tends to generate lower fluctuation and STD. However, when the interpolation density is larger than 6, the improvement is not that obvious. On the other hand, the total time for the calculation increases as the interpolation density increases. Hence, we think that the interpolation density of 5 should be suggested as the suitable parameter for taking the efficiency and stability into account.

## 4. Experiment and Discussion

### 4.1. Calibration Experiment

As mentioned above, the relationship between the focus wavelength and the displacement is not linear. In fact, a calibration experiment is usually one of the best choices to achieving the relationship. In this paper, a mirror was fixed on the linear stage (PI GmbH & Co. KG, Karlsruhe, Germany) to obtain five sets of measurements and determine the relationship experimentally. In the peak extraction algorithm, the interpolation density was set to 5. The focal position of the 450 nm wavelength was considered the “zero” point of the displacement. The displacement interval of the linear stage was 0.5 μm and the moving speed was 1 μm/s. In addition, the integration time of the spectrometer was set as 0.005 s. Thus, the mirror’s moving distance during the integration time is 0.005 μm, which is only 1% of the displacement interval, causing little adverse impact to the calibration results.

**Figure 6:**
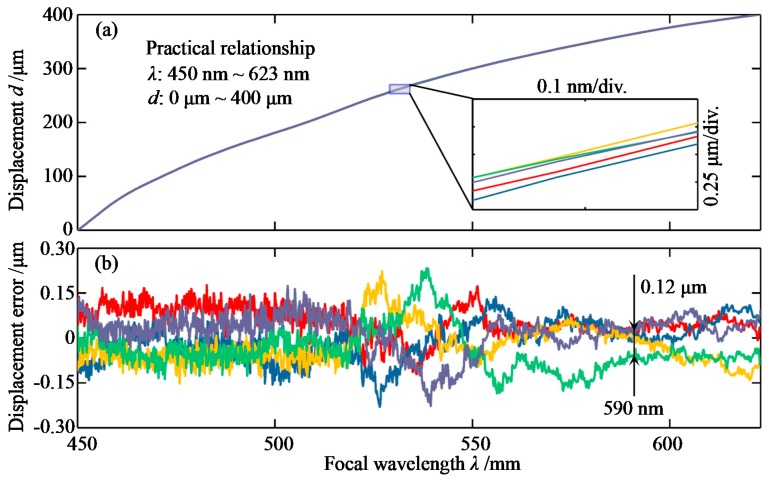
Calibration experimental results: (**a**) Practical relationship between wavelength and displacement; (**b**) Displacement error vs. the focal wavelength.

The experimental results are shown in Figure 6. We can see the displacement increases along with the focal wavelength, showing few differences with the theoretical relationship in Figure 2c. The focal wavelength ranges from 450 to 623 nm with a measurement range of 400 μm. As is known, dispersion is closely related to the refractive index of the glass. According to the optical laws, the refractive index has been expressed by many researchers without a unified formula, i.e., Cauchy, Hartmann, Conrady, and others, and the focal length is also determined by the nonlinear imaging formation of the optical system. Therefore, we think it is quite reasonable that the trend between the displacement and the focal wavelength is nonlinear. Hence, in the practical measurement, the displacement is determined from the calibration curve by interpolation. Moreover, the five curves are too close to each other to separate, so the displacement error is shown in Figure 6b to show the calibration repeatability. The measurement results verify that the system has a high calibration repeatability of about ±0.3 μm in the full measurement range of 400 μm. The displacement error is partly caused by the uncertainty of the linear stage, which was studied in our previous research [27]. The best repeatability is about 0.12 μm at the wavelength of 590 nm. We believe this is due to the SNR distribution of the LED light source, i.e., the intensity at 590 nm is always very high and generates a relatively high SNR. In conclusion, the system can provide calibration repeatability of ±0.3 μm in the full measurement range of 400 μm, which corresponds to ±0.075%.

### 4.2. Stability Experiment

**Figure 7:**
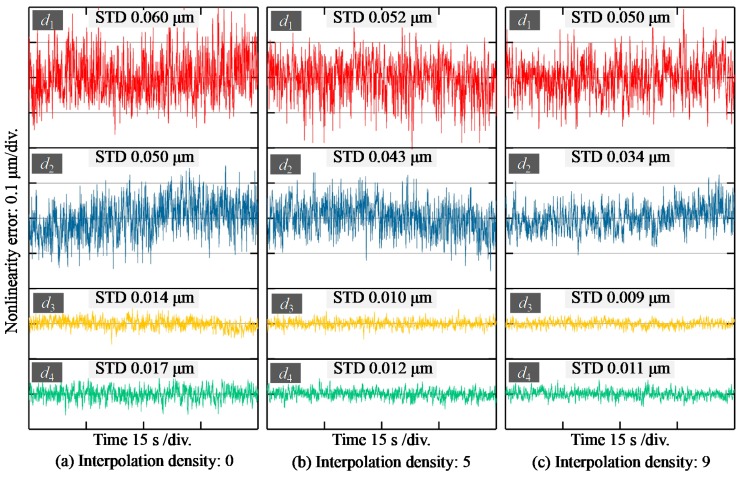
Stability at different displacements with different interpolation densities (*d*_1_ = 30 μm, *d*_2_ = 220 μm, *d*_3_ = 360 μm, *d*_4_ = 390 μm).

Figure 7 shows the stability of the system. The mirror was fixed on the stage at four different displacements within the measuring range of 400 μm in the calibration experiment, i.e., 30, 220, 360, and 390 μm relative to the focal position of the 450 nm wavelength. Figure 7a–c were obtained at interpolation densities of 0, 5, and 9. It can be seen that with the increase of interpolation density, the standard deviation (STD) reduces accordingly. However, the improvement is not that obvious between Figure 7b and Figure 7c. In practical terms, the larger the interpolation density, the longer the calculation time, which will influence the measuring efficiency. Thus, the interpolation density of 5 is recommended for this system to balance the conflict of stability and measuring efficiency. On the other hand, the figure clearly shows that the standard deviation (STD) is no larger than 0.01 μm at the displacement of 0.36 mm, and the biggest STD is about 0.052 μm at the smallest displacement tested. This is mainly caused by the spectrum of the white light source, whose highest intensity occurs at the wavelength of 590 nm, producing a better SNR at the focal position of 0.36 mm. In addition, it should be noted that although the modified algorithm can improve the stability of data processing by an order of about 20%, more optimization of the light source SNR is required if we are to further improve the stability.

### 4.3. Sample Measurement

**Figure 8:**
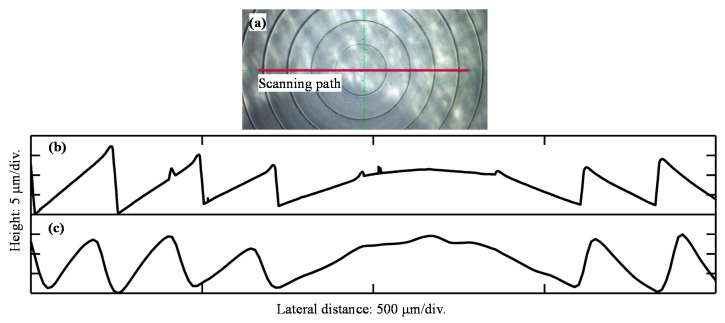
Fresnel lens section profile. (**a**) Scanning path; (**b**) P-7 test result; (**c**) CCT test result.

The Fresnel lens is a novel element that offers light focusing and collimation without the bulk associated with traditional lenses, which reduces its weight considerably. It contains a series of annular steps effectively dividing the continuous surface of a standard lens into a set of surfaces of the same curvature. Hence, the profile of the grooved surface has important implications for the focal length and transmission of the lens. We used the CCT system to measure a Fresnel lens (Thorlabs, FRP125, Newton, NJ, USA) to assess the system’s capacity for the profiling of microstructures. The scanning speed was set as 0.1 mm/s. The nominal lens groove spacing is about 0.25 mm, and we employed the P-7 profiler (KLA Tencor, Milpitas, CA, USA) to provide a reference with a scanning speed of 0.02 mm/s. The measured Fresnel lens section profiles are shown in Figure 8.

It can be seen that the two surface profiles are similar, and the groove spacings of both measurements are close to 250 μm. However, the exact height is not the same mainly because the P-7 profiler used a diamond probe with an included angle of 60°, which was easily interfered by the large slope angle at the descending edge during the scanning [28]. Besides, the lateral resolution of the CCT system is limited by the focal spot size, leading to a smoother peak and valley, which may also result in the different heights. Furthermore, the measuring position cannot be exactly adjusted between the two separate tests. As shown in Figure 8a, the scanning path of the P-7 profiler is along the red line, while that of the CCT system may deviate from the line by several microns to produce an Abby error. These systematic differences led to the phenomenon that the heights of the two profiles are not exactly the same. However, CCT and P-7 can both measure the slightly sloping surface well enough to provide similar profiles with close groove spacings. Furthermore, the scanning speed of the CCT system can be set much higher than the P-7 because the optical probe must not harm the surface or be destroyed during the measurement. According to a series of experiments with different scanning speeds, we concluded that the speed of 0.1 mm/s is acceptable to describe the surface well, ignoring some small and insignificant details. In short, the CCT system’s scanning speed can reach a much faster level than that of the P-7′s scanning speed.

In conclusion, the sample measurement shows that CCT is a feasible method for the profile measurement of minute structures with some slightly steep surfaces and narrow corners. It can provide some useful information with a very high scanning efficiency, such as the periodic spacing.

## 5. Conclusions

This paper proposed an optical design strategy for the dispersion probe with a magnification of 0.3×. The detailed design targets were determined to show good properties. The outlet NA’ is about 0.45, which means the probe receives most of the reflected light within an angle of ±27°. When the fiber core diameter is 50 μm, the focus spot diameter reaches 15 μm. The centroid peak extraction strategy was modified with several virtual pixels interpolated into the pixel-to-intensity spectrum. The modified centroid algorithm was applied to reduce the focal wavelength measurement fluctuation range from 0.059 to 0.047 μm. An interpolation density of 5 was used to guarantee the measuring efficiency. With the calibration experiments, the displacement–wavelength relationship was determined with the measurement range of 400 μm for a wavelength bandwidth of 450–623 nm. The stability experiments show better system properties with the modified centroid method, achieving the best STD of 0.009 μm. Finally, a Fresnel lens was measured using the proposed CCT system, showing better adaptability for slope surface and higher efficiency than the contact profiler.

In conclusion, the proposed optical design strategy meets the multiple requirements of the dispersion probe in the high-precision CCT system. The experiments demonstrate that the CCT system can provide high feasibility and efficiency for the profile measurements.
